# Gun Shot Wound

**Published:** 1854-07

**Authors:** Ira J. Oatman

**Affiliations:** Sacramento City, California


ART. II.—*Gun Shot Wound. Three-fourths-ounce Ball in the Bladder.* By IRA J. OATMAN, M. D., Sacramento City, California.

J. H., a German, received an accidental shot from a shot gun, containing a ball of $\frac{3}{4}$ oz. weight, two inches above the hip joint and two inches below the superior crest of the ilium of the left side. The ball passed through the glutei muscles anteriorly and to the right and entered the bladder. He directly passed near a pint of blood from the penis, when it ceased flowing, but he felt pain and tenderness in pubic region with some distention. Two hours afterwards, by introducing a silver catheter, he was enabled to pass voluntarily a large tumbler of partially coagulated blood.

I drew off his urine for two days, when, and afterwards, he could pass it at will by lying on his back.

He was quite well in less than four weeks without ever passing any urine through the wound.

When he first began to be up, as he could not urinate in the erect position, I found that the ball was loose in the bladder and that it could be felt distinctly with the sound. It acted as a globular valve and closed the neck of the bladder when he was erect, but when "flat" on his back, he could urinate freely. He refused to be operated upon for the removal of the ball, and went to the mountains several months. I have not heard from him since.

